# Systemic innate myeloid responses to acute ischaemic and haemorrhagic stroke

**DOI:** 10.1007/s00281-022-00968-y

**Published:** 2022-11-08

**Authors:** Ruth Stephens, John R. Grainger, Craig J. Smith, Stuart M. Allan

**Affiliations:** 1 Division of Neuroscience, School of Biological Sciences, Faculty of Biology, Medicine and Health, University of Manchester, Manchester, UK; 2 Geoffrey Jefferson Brain Research Centre, Manchester Academic Health Science Centre, Northern Care Alliance NHS Foundation Trust, University of Manchester, Manchester, UK; 3 Lydia Becker Institute of Immunology and Inflammation, Manchester Academic Health Science Centre, University of Manchester, Manchester, UK; 4 Division of Immunology, Immunity to Infection and Respiratory Medicine, School of Biological Sciences, Faculty of Biology, Medicine and Health, University of Manchester, Manchester, UK; 5 Division of Cardiovascular Sciences, School of Medical Sciences, Faculty of Biology, Medicine and Health, University of Manchester, Manchester, UK; 6 Manchester Centre for Clinical Neurosciences, Northern Care Alliance NHS Foundation Trust, Salford, UK

**Keywords:** Stroke, Immune, Myeloid, Inflammation

## Abstract

Acute ischaemic and haemorrhagic stroke account for significant disability and morbidity burdens worldwide. The myeloid arm of the peripheral innate immune system is critical in the immunological response to acute ischaemic and haemorrhagic stroke. Neutrophils, monocytes, and dendritic cells (DC) contribute to the evolution of pathogenic local and systemic inflammation, whilst maintaining a critical role in ongoing immunity protecting against secondary infections. This review aims to summarise the key alterations to myeloid immunity in acute ischaemic stroke, intracerebral haemorrhage (ICH), and subarachnoid haemorrhage (SAH). By integrating clinical and preclinical research, we discover how myeloid immunity is affected across multiple organ systems including the brain, blood, bone marrow, spleen, and lung, and evaluate how these perturbations associate with real-world outcomes including infection. These findings are placed in the context of the rapidly developing field of human immunology, which offers a wealth of opportunity for further research.

## Introduction

Advances in the field of stroke medicine and management of cardiovascular health have led to a steady decline in stroke incidence and stroke-related mortality and morbidity in high income countries [1, 2]. In particular, new treatments like endovascular thrombectomy for the treatment of large vessel occlusion ischaemic stroke have been shown to be effective in reducing rates of disability [3–5]. Haemorrhagic stroke, whilst less prevalent than ischaemic stroke (global incidence: 37.6%, haemorrhagic; 62.4% ischaemic), accounts for comparatively higher morbidity burden, and is hampered by lack of therapeutic interventions [1, 6]. Intracerebral haemorrhage (ICH) is the most prevalent form of haemorrhagic stroke, and is caused by a bleed within the brain tissue or parenchyma, whereas subarachnoid haemorrhage (SAH) is precipitated by a bleed into the subarachnoid space within the meninges [1, 7]. Challenges in the treatment of ischaemic and haemorrhagic stroke centre on reducing mortality rates associated with secondary brain injury and infection and, as survival rates improve, reducing the impact of disability and long term sequelae including cognitive impairment [8–11]. Amounting evidence suggests that immune dysregulation may be a target to improve brain injury, recovery, and outcome with respect to these challenges.

The activation of the peripheral innate immune system coupled with systemic inflammation is a fundamental feature of the immune response to stroke, contributing to both secondary brain injury and repair [12–14]. However, in combination with lymphopenia, aberrant systemic inflammation and innate immune dysfunction can leave the host vulnerable to secondary infection [15–17]. Thus, the innate immune system has two distinct overlapping roles in stroke pathophysiology, first in the inflammatory response to brain injury and second in the maintenance of host defence. This review will focus on profiling the myeloid arm of the innate immune system, delineating specific roles for neutrophils, monocytes, and dendritic cells (DCs) in response to ischaemic and haemorrhagic brain injury and maintenance of host immunity. Discussion will focus on human stroke immunology, and place findings within the context of new conceptual frameworks for myeloid cell immunology in the single cell era.

## The CNS

### Priming the environment: early stages of brain injury

Acute cerebral ischaemia triggers the rapid activation of inflammation in response to inadequate perfusion, and in the case of haemorrhagic stroke, presence of extravasated blood in the brain parenchyma, subarachnoid space, and cerebrospinal fluid (CSF) [18–21]. Immunogenic inflammatory activity is concentrated within the peri-infarct and peri-haematomal tissues in the acute period following stroke, and is mediated by the activation of tissue resident macrophages/microglia, endothelial cells and astrocytes [11, 22–24]. These brain resident cells sense and respond to local damage associated molecular patterns (DAMPs) released by dead and dying cells, via the induction of a repertoire of inflammatory pathways through toll-like receptor signalling [24–27]. Production of inflammatory cytokines, chemokines, and proteinases alongside the upregulation of endothelial cell adhesion molecules, primes the central nervous system (CNS) and vasculature for the entry of peripheral leukocytes [28–32]. Neutrophils and monocytes are amongst the first leukocytes to invade the inflamed CNS, where they populate meningeal, parenchymal, and vascular tissues [33–39]. Myeloid cell infiltration peaks around 1–5 days post-injury [36, 40–43] before declining steadily over the course of a week, at which point B and T cells become established in brain tissue [44, 45]. This critical time period (1–5 days) during the subacute stage coincides with the peak of systemic inflammation, development of nosocomial infections, and early mortality as seen in acute ischaemic and haemorrhagic stroke patients [46–52].

### Myeloid cell recruitment to the CNS

#### CNS anatomy

Leukocyte entry to the CNS following stroke is tightly regulated by a network of specialised neurovascular barriers. There are three main gates by which leukocytes may infiltrate the CNS from the peripheral blood: the blood–brain barrier (BBB) in the brain parenchyma, the blood-meningeal barrier in the subarachnoid space, and the blood-CSF barrier in the choroid plexus [53]. The brain parenchyma during homeostasis is impermeable to peripheral blood leukocytes existing in a state of ‘immune privilege’. At steady state, the parenchyma is populated only by resident myeloid cells: the microglia and perivascular macrophage [53]. By contrast, the meninges and choroid plexus comprise a broad range of myeloid populations including border associated macrophages, monocytes, DCs, and granulocytes [54, 55].

Following stroke, myeloid cells from the periphery accumulate within meningeal, parenchymal, and choroid plexus tissues [35, 36]. Emerging evidence from human and rodent studies suggest that peripheral myeloid cells exhibit divergent activation states in response to stroke, which can vary across different anatomical niches [56, 57]. In contrast, the myeloid response to ischaemic and haemorrhagic stroke exhibits several core overlapping elements, suggestive of a level of redundancy in the response to inflammatory brain injury [35, 58–60].

#### Brain parenchyma

Neutrophils and monocytes infiltrate the brain parenchyma in both experimental ischaemic and haemorrhagic stroke, mediated by the loss of BBB integrity in response to inflammation [30, 61, 62]. Extravasated parenchymal populations of neutrophils and monocytes have also been observed in ischaemic stroke patients post-mortem, confirming their clinical relevance [33–39].

Leukocyte chemotaxis is mediated by a compromised BBB, which acts alongside cytokines and chemokine gradients to attract myeloid cells to the brain parenchyma. Chemokine signalling is an essential regulator of myeloid cell recruitment to the CNS and distribution within the brain parenchyma. Neutrophil chemotaxis is supported by a plethora of chemokines including CXC chemokine ligand (CXCL)1, C–C chemokine ligand (CCL)5, CXCL12, and CXCL13 which are rapidly increased in response to experimental stroke [63]. In humans, neutrophil chemokines are upregulated locally in CNS compartments following ischaemic stroke. CXCL4 and CXCL7 are increased in the cerebral circulation relative to the systemic circulation [64], whereas CXCL5 is increased in the CSF compartment within 24 h [65]. Neutrophil recruitment via CXCL1 is differentially regulated by interleukin (IL)-17^+^ γδ T cells, which promote CXCL1 production by astrocytes in murine models of ischaemic stroke [66]. Notably, increased CXCL1 levels have been reported in the CSF samples of ischaemic stroke patients, supporting a potential translational role for the chemokine in human subjects. [67]

CC receptor (CCR)2 /CCL2 signalling is indispensable for monocyte chemotaxis, as pharmacological or genetic inhibition of CCR2 signalling prohibits monocyte entry to the brain following experimental ischaemic and haemorrhagic stroke [29, 42, 43, 68, 69]. CCR2 signalling therefore appears to be a common mechanism by which myeloid cells are recruited to the brain following ischaemic and haemorrhagic stroke [30, 70]. In response to ischaemic stroke, monocyte distribution amongst the infarct and peri-infarct tissues appears to be regulated by the CXCL12/CXC receptor (CXCR) 4 signalling axis [56]. Retainment of monocytes within the peri-infarct tissue is observed following selective deletion of monocytic CXCR4, and associated with alternative activation programmes in brain microglia and macrophage resulting in poor outcome [56]. Therefore, monocyte localisation within the brain parenchyma, as dictated by chemokine signalling, appears to be important in the activation of both brain resident and recruited myeloid populations.

#### Other CNS tissues

The brain parenchyma does not represent the sole destination for recruited myeloid cells following stroke. Intravascular and extravascular neutrophil populations may be observed in the mouse and human brain following ischaemic stroke, throughout the brain parenchyma, perivascular spaces, and leptomeninges [36]. In human patients, neutrophil extracellular trap (NET) + neutrophil infiltrates have been found in clipped aneurysms, surgically evacuated haematoma and embolic infarct tissue, placing them at the very focal point of stroke aetiology [71–73]. Similarly, a recent single cell-RNA sequencing (scRNA-seq) study profiling the cellular makeup of arteriovenous malformations (AVM) in human patients discovered a pathogenic glycoprotein Nmb (GPNMB) + monocyte subset associated with smooth muscle cell death and AVM rupture, precipitating brain haemorrhage [74].

Myeloid localisation within meningeal and CSF tissues is of particular relevance to SAH due to the extravasation of blood into the subarachnoid space. Neutrophils, monocytes, and DCs have all been observed to accumulate in the CSF of aneurysmal SAH patients, and elevated neutrophil counts associated with cerebral vasospasm [75, 76]. Thus, it appears that neutrophils in the CSF compartment are relevant in the pathogenicity of secondary brain injury [75, 77]. Mechanistically, neutrophil infiltration of the meninges appears to be mediated by myeloperoxidase, as has been demonstrated in mouse models of SAH [78].

Myeloid cells access and populate the CNS over multiple barrier and anatomical sites following stroke. Spatio-temporal profiling of relationships between recruited immune and brain resident cells across different niches will be integral in elucidating the specific roles of myeloid cells in brain injury and repair [79]. The molecular pathways regulating myeloid cell trafficking to different CNS regions and local cues that prime specific functions require further study in the field of stroke.

### Myeloid response to stroke

#### Neutrophils

Neutrophils co-ordinate multimodal responses to stroke injury, contributing to inflammation, parenchymal, and vascular injury over the acute phase of recovery [34, 80, 81]. Several lines of evidence implicate neutrophil effector functions in BBB compromise and inflammation through the production of matrix metalloproteinase (MMP) 9, proteolytic enzymes, reactive oxidative species (ROS), and NETs [12, 35, 82]. On the macroscopic level, neutrophils contribute to the no-reflow phenomenon in ischaemic stroke, where neutrophil aggregates impede capillary micro-perfusion [83]. In mouse models of SAH, neutrophil depletion prior to injury prevents later vascular narrowing, a marker of vasospasm and mechanism related to delayed cerebral ischaemia (DCI) [77]. These findings emulate results from clinical studies linking CSF neutrophil levels with delayed cerebral ischaemia in aneurysmal SAH patients [75].

By contrast, neutrophils may also exhibit neuroprotective functions in response to stroke. Neutrophils expressing markers of alternative activation including the chitinase marker Ym1 and the phagocytic mannose receptor CD206 have been observed in mouse models of permanent cerebral ischaemia [84]. Neutrophil Ym1 expression and clearance from ischaemic tissue were increased by the peroxisome proliferator-activated receptor-γ (PPARγ) agonist rosiglitazone, and treatment was linked to a latent neuroprotective effect [84]. In the setting of experimental ICH, interleukin (IL)-27 mediated neutrophil lactoferrin production has been suggested to have a protective role in the sequestration of toxic iron products from the haematoma [33, 85]. Neutrophil depletion prior to ICH is protective against brain injury [81]; however, neutrophil depletion post injury leads to further neurological and functional damage; thus, time appears to be a critical factor in determining the neutrophil role in ICH [33].

#### Monocytes

CCR2^+^Ly6C^hi^ classical monocytes (CD14^+^CD16^−^ in human) are the main effector subset responsible for the monocyte response, and are rapidly mobilised from the circulation in response to experimental brain injury [39, 42, 56]. Fate mapping experiments in mouse models of cerebral ischaemia describe the margination of Ly6C^hi^ monocytes in the peri-infarct region, followed by the rapid differentiation of cells into a macrophage-like phenotype, via the downregulation of Ly6C and CCR2 and upregulation of the macrophage marker F4/80 [56, 86]. During the differentiation process, monocyte derived cells (MDCs) adopt divergent polarisation states, with mixed pro- and anti-inflammatory phenotypes. Fate mapping of bone marrow–derived Ly6C^hi^ monocytes in the post-ischaemic brain reveals upregulation of markers of alternative activation including Arg1, Ym1, and CD163, 24 h after ictus [86]. Sorted CD45^hi^ MDCs from the post-ischaemic brain show upregulation of genes involved in angiogenesis, efferocytosis, antigen-presentation, and anti-inflammatory factors [59, 87]. Yet, bone marrow–derived MDCs also express a core inflammatory gene signature following ischaemic stroke, through the induction of type I interferon (IFN), inducible nitric oxide synthase (iNOS), IL-1β, and IL-6 genes and retain capacity to produce IL-1β and tumour necrosis factor (TNF)α ex vivo [37, 56, 59, 88]. Similar roles relating to inflammatory cytokine production, phagocytosis, and antigen presentation have been recently profiled for MDCs isolated from the mouse ICH brain, [58] though relatively little is known of the monocyte response in SAH [39].

In vivo, the monocyte contribution to brain injury, inflammation, and neurological recovery has been profiled by selective Ly6C^hi^ monocyte depletion and targeting of CCR2 signalling pathways. Several studies describe neuroprotective roles for Ly6C^hi^ monocytes in secondary brain injury and inflammation, prevention of haemorrhagic transformation, and in the promotion of functional recovery in acute models of cerebral ischaemia [29, 43, 68, 69]. Nevertheless, inhibition of Ly6C^hi^ monocyte recruitment has been shown by others to have pathogenic or even neutral effects [89, 90]. Along similar lines, Ly6C^hi^ monocyte depletion was protective against brain injury and improved function in mouse models of ICH [42]. Therefore, both in vivo and ex vivo, classical monocytes adopt divergent roles in the response to ischaemic and haemorrhagic brain injury.

#### Dendritic cells

DCs, like monocytes, migrate to the injured brain and participate in local inflammation following experimental ICH and ischaemic stroke [91]. Depletion of brain CD11c^+^ cells have previously been shown to occur following treatment with neuroprotective therapies in experimental stroke, suggesting a role in brain injury [92]. In a preclinical model of ischaemic stroke, broad inhibition of myelin specific T cell autoreactivity reduced DC levels and attenuated brain injury, suggesting antigen presentation is significant in the priming of T cell responses in experimental stroke [93]. There is also evidence that DCs prime antigen independent responses, as neutrophil recruitment to the ischemic brain is driven by DC IL-23 activation of IL-17 γδT cells [94]*.* In human patients, conventional (cDC), plasmacytoid (pDC), and DC-T cell clusters have been identified in the ischemic and haemorrhagic brain post-mortem dispersed amongst vascular and non-vascular tissues [95].

### Assessment of the myeloid response in the single cell era

The numerous roles adopted by myeloid cells in response to acute stroke suggest conceptual frameworks based upon pro- and anti-inflammatory functional dichotomies are somewhat over-simplified. In support of this notion, monocytes and neutrophils often display mixed pro- and anti- inflammatory phenotypes at overlapping time points following stroke [59, 84]. Thus, myeloid cells appear to exhibit phenotypic and functional heterogeneity across a single population in response to brain injury.

Cell heterogeneity ascribes differences in phenotype and function across a single population, and can explain why one cell population may appear to assume many different roles in the response to stroke. Heterogeneity can be a consequence of differences in terms of cell ontogeny (tissue resident vs. recruited), localisation (parenchymal vs. vascular, core vs peri-lesion), and the time-point profiled following injury (inflammation vs. resolution phase).

Research at the single cell level is an important tool to study heterogeneity across a single population, and studies of this kind have provided novel insights on the role of macrophages in neuroinflammation [57, 96–98]. In mouse models of acute cerebral ischaemia and human ischaemic stroke, Beuker et al. [57] utilised scRNAseq to profile myeloid responses across the brain parenchyma and arachnoid pia. They identified a unique subset of parenchymal macrophages of mixed monocyte/microglial origin (SAMC), defined by the expression of a gene signature related to lipid metabolism and myelin phagocytosis, which was later functionally validated in vivo. The identification of a mixed monocyte/microglial subset is compelling, as it suggests that the parenchymal microenvironment following ischaemic stroke is capable of instructing macrophage phenotypes on cells of different haematopoietic origins. Lipid droplet rich microglia have since been identified in the aged mouse brain under steady state, and related to enhanced inflammatory type I IFN responses and poor outcome in experimental cerebral ischemia [96, 99].

Notably, the SAMC population appeared to have genetic overlap with (neuro) degeneration-associated microglia (DAM), previously identified in animal models of experimental autoimmune encephalitis (EAE), amyotrophic lateral sclerosis (ALS), and Alzheimer’s disease [97, 98, 100]. The myeloid compartment of the CNS has been extensively profiled in the setting of EAE, leading to the discovery of a novel disease associated *Cxcl10* + monocyte subset within the inflamed CNS [101]. Critical regulators of the monocyte to phagocyte transition and inflammatory function, in EAE, have also been identified for IFNγ and granulocyte macrophage–colony stimulating factor (GM-CSF) respectively [102]. It remains to be discovered whether similar frameworks governing monocyte/macrophage biology exist in the setting of acute ischaemic and haemorrhagic stroke.

One prevailing question in the field of stroke immunology is the extent to which myeloid cell ontogeny, versus lived experiences in the brain tissue, dictates resultant cell phenotypes and functions. With this in mind, we next endeavour to discuss the immune response to stroke in the periphery, detailing the myeloid response across different organs and tissues.

## The periphery

### Neuroimmune cross-talk: anatomical organisation

**Fig. 1:**
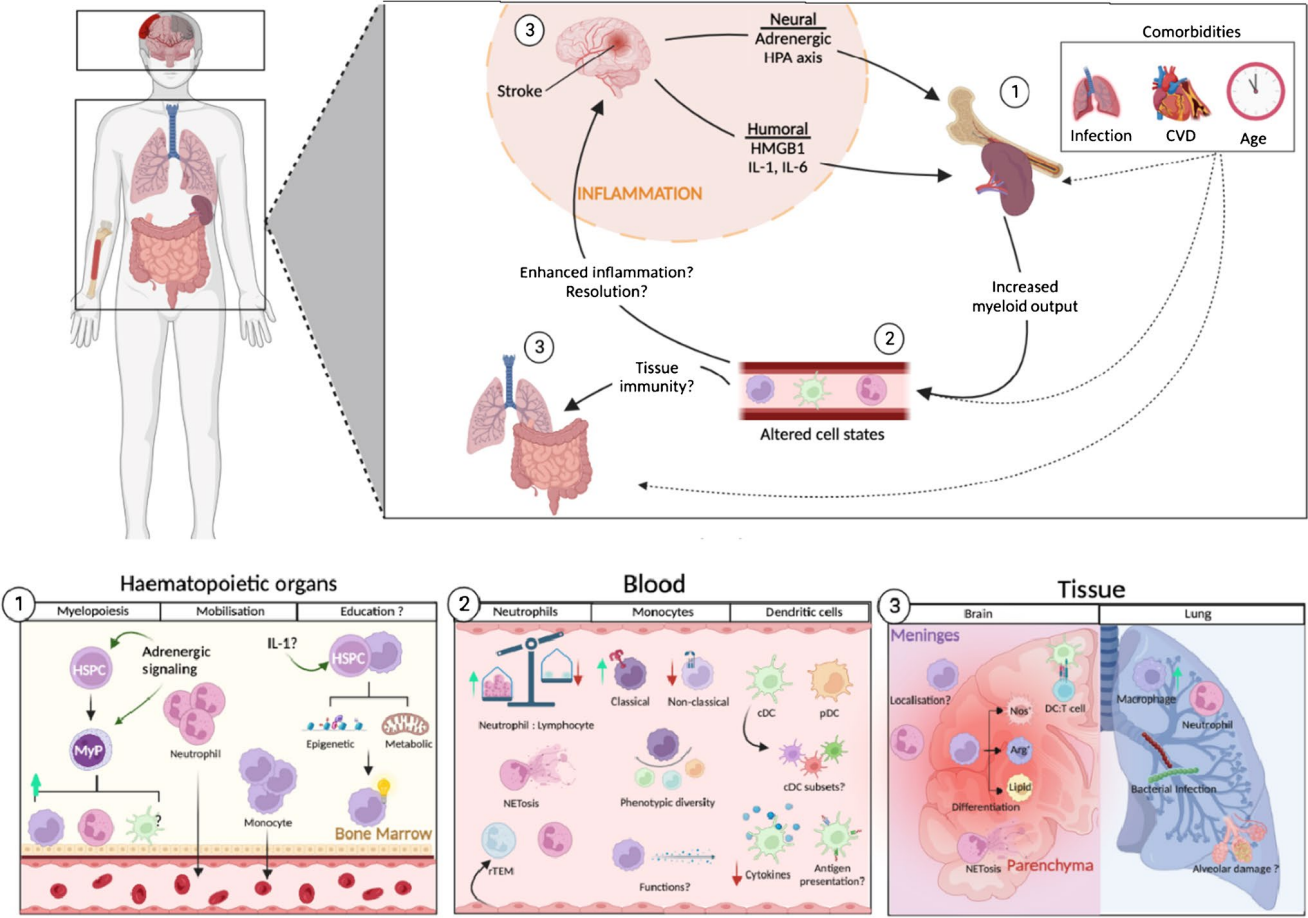
The multi-organ myeloid response to acute ischaemic and haemorrhagic stroke. Ischaemic or haemorrhagic brain injury engages emergency myelopoiesis and mobilisation of myeloid cells from tissues, through neurogenic and humoral signalling pathways (1). The bone marrow adjusts haematopoietic outputs to meet demand increasing the levels of circulating neutrophils, monocytes, and DCs, which exhibit differential phenotypes and functions in response to systemic inflammation (2). Circulating myeloid cells are delivered to distal tissues, shaping local immune responses to brain injury and infectious challenge (3). Comorbidities such as cardiovascular disease, infection, and age may modulate the scale and responsiveness of myeloid immunity to brain injury. Question marks represent proposed hypothetical pathways and avenues for further research. cDC, conventional dendritic cell; CVD, cardiovascular disease; DC, dendritic cell; HMGB1, high-mobility group box protein 1; HPA, hypothalamic pituitary axis; HSPC, haematopoietic stem cell; IL-, interleukin; MyP, myeloid progenitor; NET: neutrophil extracellular trap; pDC: plasmacytoid,,dendritic cell; rTEM, reverse transmigrated neutrophil. Created with BioRender.com

At steady state, myeloid cells circulate freely in the peripheral blood and lymph and form marginated pools in several immunological organs including the bone marrow, spleen, liver, and possibly the lung [103–108]. In response to stroke, the number of circulating myeloid cells increases dramatically, [109–113] driven by the mobilisation of marginated populations and the induction of emergency myelopoiesis in the spleen and bone marrow [41, 114, 115] (Fig. 1). These cells are funnelled to distal tissues through a network of blood and lymphatic vessels which act as a conduit for innate immune cell transport over mucosal barrier and tissue sites including the brain [116, 117].

The CNS is able to influence myeloid immunity through the induction of long-range neurogenic and humoral signalling pathways. DAMPs, molecular factors, immune cells, and self-antigen from the CNS drain into the systemic circulation and lymphatic pathways. Accumulation of these factors in secondary lymphoid organs, such as the cervical lymph nodes and spleen, perpetuates systemic inflammation [118–120]. Novel discoveries have since outlined how specialised niches within the borders of the CNS support myeloid activity in response to CNS injury and neuroinflammation. The skull bone marrow has been identified as a local haematopoietic source of myeloid cells at steady state and in injury [121], and supplies neutrophils to the meninges via microvascular channels in the dura mater following ischaemic stroke [122]. The meningeal lymphatic system located in the cribriform plate has been identified as an important immune-regulatory niche in EAE, supporting antigen presenting interactions between CD11c + myeloid and CD4 + T cells, alongside the drainage of CSF, CNS antigen, and immune cells from the inflamed CNS [123–125]. In stroke, cervical lymph nodes have an important role in the drainage of extravasated erythrocytes in SAH from the meninges, and CNS antigens including myelin and neuronal proteins in ischaemic stroke patients [119, 126].

Neurogenic control of systemic immunity is co-ordinated through the sympathetic nervous system and hypothalamic pituitary axis [114, 127, 128]. Increased sympathetic drive as measured by circulating catecholamine levels is seen in patients [129–131], and is of particular relevance in aneurysmal SAH, where sympathetic activation is associated with the development of complications relating to cardiac dysfunction, neurogenic pulmonary oedema, and hypertension [129, 132, 133]. The sympathetic nervous system innervates both primary and secondary lymphoid organs, and promotes immunogenic activity at distal tissues following stroke [114, 115]. For example, adrenergic signalling has been shown to drive myeloid differentiation programmes in the bone marrow, induce lymphocyte apoptosis in the spleen, and enhance immunosuppressive functions of liver iNK-T cells in response to ischaemic stroke [114, 134–136].

### Emergency myelopoiesis

#### Bone marrow

Systemic inflammation coupled with the activation of adrenergic signalling pathways promotes myeloid haematopoietic bias following stroke. Myeloid bias in the bone marrow is evident through increased numbers of haematopoietic stem cell (HSC), granulo-monocyte progenitor (GMP), and monocyte dendritic progenitors (MDP) in contrast to decreased common lymphoid progenitor (CLP) counts 3 days after acute ischaemic or haemorrhagic stroke [114, 115]. Excised skull flap bone marrow samples from ICH patients undergoing surgical craniotomy also show expanded populations of GMP and common myeloid progenitors (CMP) [115]. Thus, the skull may represent an important local reservoir by which myeloid cells are deployed directly to the CNS [121].

Inflammatory factors can activate toll-like, cytokine, growth factor, or adrenergic receptors expressed on bone marrow stroma and progenitor cells, promoting myelopoietic bias through the induction of specific developmental factors [137]. Following experimental ischaemia, increased Ly6C^hi^ monocyte, and neutrophil levels coincide with increased expression of the transcription factor Pu.1 in the bone marrow [114]. Pu.1 is an essential regulator of monocyte and neutrophil development through the GMP lineage [138–140]. Whilst the modulation of Pu.1 by molecular factors has not been investigated in stroke per se, increased adrenergic innervation of the bone marrow following stroke has been associated with HSC cycling and activation in mouse models of cerebral ischaemia and ICH [114, 115]. Adrenergic signalling via β3 adrenergic receptors expressed on mesenchymal stem cells, enhances HSC mobilisation through the modulation of CXCL12, and promotes myeloid differentiation [141, 142]. Soluble inflammatory factors such as type I IFNs and IL-1, which are upregulated in acute inflammation [96, 143], may also regulate bone marrow myeloid bias in stroke. Type I IFNs have been implicated in emergency myelopoiesis in mouse models of endotoxemia (sepsis) [144], and enhanced type I IFN activation in murine bone marrow myeloid cells is a feature of myocardial infarction [145]. Acute elevation of IL-1, as seen in stroke [146–149], can induce HSC proliferation and drive myelopoiesis via Pu.1 activation [150].

#### Spleen

Emergency myelopoiesis is not exclusive to the bone marrow, and is often observed in the spleen in acute inflammation. In models of myocardial infarction, adrenergic innervation of the bone marrow promotes HSC seeding of the spleen resulting in extramedullary myelopoiesis [142, 151]. Though splenic haematopoiesis within the context of ischaemic or haemorrhagic stroke has yet to be investigated, the spleen mounts a dynamic response to brain injury. Spleen contraction is seen in human ischaemic stroke and ICH patients as well as in mice following experimental ischaemia [135, 152] In mouse, splenic atrophy coincides with activation of adrenergic signalling and loss of splenic marginal zone B cells, whereas splenic macrophages exhibit dynamic micro-anatomical and phenotypic changes following cerebral ischaemia [120, 135]. Splenic atrophy may also arise as a consequence of innate cell mobilisation from marginated pools. In vivo tracing of splenocytes following MCAo in rat shows monocytes alongside NK cells and T cells traffic to the brain via the peripheral blood [153]. In patients with acute ischaemic stroke and ICH, spleen shrinkage was observed in 40% of the study cohort, within 24 h of initial injury and associated with stroke severity and systemic increase of inflammatory cytokines including IFNγ, IL-6, IL-10, IL-12, and IL-13 [152].

### Peripheral blood compartment

#### Neutrophil

Following acute ischaemic and haemorrhagic stroke, a transient increase in circulating neutrophil counts is observed [109–111]. In the clinical setting, the neutrophil to lymphocyte ratio (NLR) is commonly used as a biomarker of immunological dysfunction. In aSAH patients, NLR has been linked to stroke severity and has been identified as a prognostic biomarker of DCI and vasospasm [154, 155]. In ICH and ischaemic stroke patients, high NLR at admission is predictive of poor 90-day outcome and haemorrhagic transformation respectively [156, 157]. Further studies in ischaemic stroke patient cohorts have shown increased NLR scores strongly relate to infection, poor outcome, and mortality at 3 months [158–160]. Therefore, one may conclude that high neutrophil counts, particularly when coupled with lymphopenia, could have a detrimental role in the recovery from brain injury.

Neutrophils respond to immunologic stress by adopting a broad range of phenotypes, which reflect changes in neutrophil lifecycle and activation states [161]. Molecular cues, increased systemically in inflammation, may encourage divergent phenotypes and polarisation resulting in heterogeneity [161]. Within the peripheral blood compartment, acute inflammation is associated with the emergence of immature, senescent, inflammatory, immune-suppressive, and reverse transmigrated (rTEM) neutrophil subsets and states [110]. A recent study profiling the hyperacute neutrophil response in ischaemic stroke patients revealed neutrophils adopted senescent (CXCR4^bright^CD62L^dim^) and rTEM(CD54^hi^CXCR1^lo^) phenotypes. rTEM neutrophils are able to migrate from tissues to the peripheral blood, a process observed in mouse models of lipopolysaccharide (LPS)-induced neuroinflammation [162]. Moreover, rTEM neutrophils exhibit inflammatory functions, may redistribute to peripheral organs such as the lung following sterile injury, and inhibit T cell proliferation [159, 163, 164] processes which may contribute to impeded host immunity following acute ischaemic stroke. Notably, this study did not find evidence of immature neutrophils, and immune-suppressive neutrophils (CD16^bright^CD62L^dim^) were unchanged. With time, neutrophil phenotypes in systemic circulation and in tissues are likely to shift, reflecting ontogeny (emergency myelopoiesis), and activation state (maturation of the inflammatory microenvironment) [110].

#### Monocyte

Consistent clinical studies have shown monocytes expand in the peripheral blood in response to ischaemic and haemorrhagic stroke, and strongly associate with markers of outcome. In ischaemic stroke, elevated blood monocyte counts have been associated with stroke severity, mortality, and incidence of infection [15, 112, 165]. In ICH, studies have previously described an association between high monocyte counts on admission with mortality and poor outcome [112, 166]. Latent monocyte infiltration of the CSF in aneurysmal SAH patients has been related to DCI [76], whereas elevated levels of peripheral blood monocytes at admission have been related to vasospasm and hydrocephalus [113, 167]. Therefore, clinical findings consistently support a role for monocytes in both primary and secondary brain injury.

Stroke differentially regulates discrete subpopulations of blood monocytes. Specifically, clinical studies in ischaemic stroke and ICH have reported an expansion of classical (CD14^+^CD16^−^) and intermediate (CD14^+^CD16^+^) populations at the expense of non-classical monocytes (CD14^lo^CD16^+^) [15, 168]. Higher classical monocyte counts have been associated with poor outcome, mortality, and clinical worsening, whereas high intermediate counts are associated with protection against mortality [15, 168]. As discussed earlier, translational studies have implicated the murine classical monocyte equivalent (Ly6C^hi^CCR2^+^) as the primary monocyte effector within the brain. Expansion of circulating classical monocytes in the blood is likely a reflection of enhanced release of these cells from haematopoietic organs, and supports the ongoing recruitment of classical monocytes to the brain.

Decreased non-classical monocyte levels are associated with poor outcome and increased risk of infection [15, 168]. Depletion of non-classical monocytes from the peripheral blood is a feature of acute inflammation witnessed in sepsis [169] and COVID-19 [170], and is posited to be the result of cell death [171], vascular patrolling [172], or reflect altered monocyte differentiation kinetics. At steady state, a subset of classical monocytes remains in the blood and differentiates into intermediate followed by non-classical monocytes [173]. In conditions of immunologic stress, such as LPS driven endotoxemia, acute monocytopenia (two hours) is followed by the sequential repopulation of classical followed by intermediate and non-classical monocyte subsets (72 h) [173]. Whilst the time point sampled by previous clinical studies is too late to confirm monocytopenia in stroke, this paradigm may explain the kinetics of monocyte subset regeneration in response to systemic inflammation. Nevertheless, one cannot completely rule out the possibility of non-classical monocyte recruitment to the brain. Elevated levels of CD16^+^ monocytes are reported in CSF samples of aSAH patients, relative to a decrease of circulating CD16^+^ monocytes in the peripheral blood [76, 174]. ICH and aSAH in particular require extensive cerebrovascular remodelling in major vessels. Recruitment of non-classical monocytes to the cerebral vessels could support this given their role as vascular patrolling cells [172]. In support of this view, a recent study in a model of mild traumatic brain injury (TBI) outlined a specific role for non-classical monocytes in the promotion of angiogenesis in the meningeal vasculature [175].

#### Dendritic cell

Both ischaemic and haemorrhagic stroke patients exhibit characteristics of impairments to the DC compartment post-stroke. Consistent reports have described decreased levels of total DCs, cDCs, and pDC levels in the peripheral blood of ischaemic stroke and ICH patients [95, 176]. Moreover, cDCs, pDCs, and DC-T cell clusters have been identified in the ischaemic and haemorrhagic brain post-mortem, and pDCs increase in the CSF of aSAH patients at a late time point [76, 95]. Although the precise mechanisms underlying decreased levels of DCs within the peripheral blood have not been identified, the presence of DCs in the brain may be indicative of recruitment of cells from the periphery and a role for them in brain injury.

### Myeloid cell function and training

In addition to phenotypic changes, myeloid cells may also exhibit impaired innate or adaptive functions following acute ischaemic or haemorrhagic stroke. Accordingly, peripheral blood neutrophils display features of a hyper-inflammatory functional state following acute ischaemic stroke as marked by decreased CD62L expression, increased CD11b expression, and enhanced NETosis, ROS production, and levels of elastase [110].

In humans, circulating monocytes exhibit hallmarks of functional deactivation post-stroke which is associated with stroke-associated infection. Expression of the antigen presenting molecule HLA-DR on monocytes is diminished in stroke patients and correlates with incidence of stroke associated infection [15, 177]. Likewise, increased monocytic expression of Tim-4, a molecule with T cell costimulatory capacity, correlated with poor outcome in ischaemic stroke patients [178]. Both HLA-DR and Tim-4 are critical mediators of T cell activation in response to antigen, suggesting the initiation of adaptive immunity may be impaired in stroke. Monocyte innate effector functions also appear to be compromised, as in vitro, monocytes isolated from stroke patients exhibit hallmarks of a refractory state following recurrent stimulation with LPS [179]. DCs also appear to have impaired innate effector functions following stroke. DCs isolated from aSAH patients had impaired cytokine production in response to TLR stimulation compared to non-stroke controls [176]. Other parameters of DC function remain to be fully characterised; for example, it is unclear whether specific type 1 or type 2 responses are promoted by DCs or whether DC subsets act in a tolerogenic manner within the context of stroke.

#### Trained immunity

Arguably, the presentation of differential functions of myeloid cells when primed in vitro along with altered functional phenotypes ex vivo is suggestive of trained immunity. Trained innate immunity is driven by epigenetic or metabolic reprogramming, which results in altered immune cell responses to subsequent immunogenic challenge [180, 181]. This training can either enhance immune responses, or can impair them, resulting in dysregulated immunity and further pathogenicity [181]. Indeed in stroke, tolerogenic functions could be protective, reducing systemic inflammation and risk of autoimmunity directed towards self-antigen [119, 182]. On the other hand, innate immune paralysis and senescence may leave the host vulnerable to infection, if myeloid functions are overtly suppressed [177, 179].

Epigenetic or metabolic evidence of innate immune reprogramming in response to stroke is yet to be discovered. Yet, several prominent regulators of the systemic inflammatory response to ischaemic and haemorrhagic stroke, including High mobility group box protein 1 (HMGB1) and catecholamines, have the potential to induce training in other models of inflammation [183, 184]. Moreover, haematopoietic organs (bone marrow and spleen) can act as integrative educational hubs for innate cell training. In the context of stroke, the spleen modulates the protective effects of LPS pre-conditioned monocytes following tMCAo. Ex vivo LPS ‘priming’ led to the accumulation of neuroprotective monocytes in the brain and meninges following adoptive transfer to a tMCAo mouse. The neuroprotective effect was lost in splenectomised mice, suggesting the spleen was essential in mediating this benefit [185].

Long-range signalling in inflammation may also prime myeloid cells early on in development. In the setting of myocardial infarction, long-range signalling instructs type I IFN programs in bone marrow monocyte and neutrophil populations, which are maintained by cells infiltrating the ischaemic myocardium [145]. In this case, the cells are already ‘primed’ to initiate type I IFN programs upon tissue entry. In a mixed model of experimental TBI with infection, Type I IFN programmes initiated in myeloid cells in response to infectious challenge, disrupted vascular repair in the meninges and brain parenchyma following experimental TBI [186]. Thus, infection and brain injury appear to dictate divergent immunological programs, with infection driven type I IFN activation detrimental to neurovascular repair and recovery. Nevertheless, the debate surrounding long range vs. local signals on informing cell fate and function continues. Longitudinal immune sequencing of monocytes and neutrophils in haematoma and peripheral blood samples from ICH patients revealed divergent genetic profiles between circulating and haematoma populations suggesting the brain microenvironment dictates terminal cell activation states irrespective of peripheral phenotype [187].

#### Lung immunity and stroke

Brain injury may modulate innate cell function in distal organs, rendering them vulnerable to infectious challenge. In stroke, attention has recently focused on the lung and the gut, two immunogenic organs central to the maintenance of host defence. The gut-immune brain axis with respect to stroke and other neurological disorders has been the subject of extensive review [188–190]**.** though studies examining the lung are comparatively few. Nosocomial pneumonia is the most prevalent infection reported amongst ischaemic stroke patients, and is linked to dysphagia, stroke severity, and immune dysregulation [191–193]. Experimental cerebral ischaemia increased macrophage and neutrophil counts in bronchiolar lavage fluid (BALF) 24 h after ictus, consistent with upregulated gene expression of the inflammatory cytokine IL-1β [194]. The lungs may also be subject to inflammatory injury themselves, as marked by alveolar damage and oedema 24 h following experimental cerebral ischaemia [195]. Acute lung injury and acute respiratory distress syndrome contribute to poor outcome in SAH patients [196, 197]. In aged mouse models, increased lung bacterial burden following stroke was associated with increased inflammation, and impaired neutrophil chemotaxis and bactericidal functions [198].

## Conclusion

The pre-clinical and clinical studies presented in this review outline a role for the myeloid arm of the peripheral innate immune system in the response to acute ischaemic and haemorrhagic stroke. Preclinical studies in rodent models of stroke, supported by clinical research, have revealed how myeloid cells adapt across different CNS compartments and niches by changing phenotype and function. For example, it is clear that inflammation functions as a pre-requisite for the initiation of repair programmes in myeloid cells. Identifying the critical regulators of these phenotypes, functions, and anatomical distribution of myeloid cell post-stroke represents an opportunity for therapeutic intervention.

Research focusing on the peripheral immune system has built a picture of disrupted immunity across multiple organ systems, including the bone marrow, spleen, blood, and lung. Each of these tissues exhibit a dominant myeloid response in the acute stage of stroke, often at the expense of adaptive immunity. Evidence from clinical studies suggests this myeloid bias is related to stroke severity and is associated with key measures of outcome, including stroke associated infection. However, the long-term consequences of this response are still somewhat unclear, as is how the innate immune system primes adaptive immunity and translates into immune-suppression.

Whilst the literature paints a rich picture of the myeloid response in ischaemic stroke, haemorrhagic stroke including both ICH and SAH subtypes remains comparatively under-researched. It is likely some level of redundancy exists between ischaemic and haemorrhagic stroke in terms of the peripheral immune response and systemic inflammation. Yet further research is critical in understanding how haemorrhagic stroke drives systemic immune dysregulation, and the differential requirements of the myeloid response in resolving haemorrhagic brain injury compared to ischaemic stroke.

The field of human stroke research may gain from recent technological breakthroughs in the field of human immunology. As immunology moves into the single cell era, advancements in the fields of flow cytometry, transcriptomics, proteomics, metabolomics, and data analytics present new opportunities for research in human stroke immunology. On the other end of the scale, epidemiological research driven by large databases and supported by big data analytics will be integral in researching ischaemic and haemorrhagic stroke across entire populations. Expanded human research could encourage translational research in the reverse. That is, research questions identified in human could be back-translated using relevant mouse models to test hypotheses and identify targetable molecular pathways for therapeutic intervention.
